# Inflammatory cytokines and combined biomarker panels in pancreatic ductal adenocarcinoma: Enhancing diagnostic accuracy

**DOI:** 10.1371/journal.pone.0221169

**Published:** 2019-08-15

**Authors:** Deirdré Kruger, Yandiswa Y. Yako, John Devar, Nicola Lahoud, Martin Smith

**Affiliations:** 1 Department of Surgery, School of Clinical Medicine, Faculty of Health Sciences, University of the Witwatersrand, Johannesburg, South Africa; 2 Hepato-Pancreatico-Biliary Unit, Department of General Surgery, Chris Hani Baragwanath Academic Hospital, Johannesburg, South Africa; University of Nebraska Medical Center, UNITED STATES

## Abstract

**Background:**

Early diagnosis of pancreatic ductal adenocarcinoma (PDAC) is challenged by the absence of accurate early diagnostic and prognostic biomarkers. CA19-9 is the established, diagnostic tumour marker in PDAC, despite its limitations. Effective primary screening using circulating biomarker panels have only been considered in a handful of studies and we investigated whether combinations of inflammatory cytokines and angiogenic factors in multivariate logistic models could facilitate earlier diagnosis in our South African setting.

**Methods:**

Plasma levels of 38 cytokines and angiogenic factors were measured in 131 Black South African patients, 85 with PDAC, 25 with benign biliary pathology (BBP) and 21 benign non-HPB controls (BC), by use of human magnetic multiplex screening assays. Multivariate biomarker panels were developed by identifying the top performing biomolecules from univariate logistic regression. Receiver-operator characteristic (ROC) curves and area under the ROC curve (AUC) are reported.

**Results:**

Classification modelling to distinguish PDAC patients from BC showed that a panel of CA19-9 and CXCL10 (IP-10) demonstrated improved diagnostic power over CA19-9 alone (AUC = 0.977 vs. AUC = 0.807, p-value = 0.001). A combined panel including age, BMI and IL-15 showed significant diagnostic power in discriminating PDAC from BBP (AUC = 0.952, p < 0.0001). Finally, a combined panel of IL-8, IL-15 and gender demonstrated diagnostic accuracy (AUC = 0.830, p < 0.0001) in distinguishing PDAC in the presence of jaundice from benign controls with either jaundice, choledocholithiasis or common bile duct injury.

**Conclusions:**

Combined biomarker panels improve diagnostic accuracy in PDAC. In addition to CA19-9, cytokines CXCL10, IL-8 and IL-15 are strong additions to diagnostic biomarker panels in PDAC in Black South Africans.

## Introduction

Pancreatic ductal adenocarcinoma (PDAC) is predicted to become the second leading cause of death due to malignancy in the US among both men and women by 2020 [1, 2]. Most PDAC patients are asymptomatic until their aggressive disease becomes more advanced, and approximately half of patients present with metastatic disease. In recent decades there has been little improvement in the survival rate of PDAC patients, with the five-year survival rate below 5% and surgical resection, the only chance for potential cure, only an option in 10–20% of affected, eligible patients. Despite treatment advancements demonstrating significant survival advantages, survival rates remain dismal for locally advanced disease and metastatic and metastatic patients [3–6].

During the development and progression of cancer, there is a crosstalk between cancer and immune cells [7]. PDAC is characterised by increased levels of inflammatory cytokines and angiogenic markers, which play critical roles in its development and progression [8, 9]. Although the immune system initially identifies and eliminates aberrant or cancer cells through inflammatory pathways, the affected malignant cells develop protective mechanisms against host-immunity, a phenomenon called immune-editing. Moreover, the strong desmoplastic reaction in the tumour environment of PDAC reflecting the activation of pancreatic stellate cells is a hallmark of this disease, resulting in more than 50% of the tumour being covered by a dense fibrous stroma [10]. The latter further complicates immune cell infiltration and delivery of anticancer treatments [11, 12].

In addition to the complex pathophysiology, early diagnosis of PDAC is challenged by the absence of accurate diagnostic biomarkers. Where the role of CA19-9 has long been established as a diagnostic tumour marker in PDAC, its individual power to accurately diagnose PDAC from benign disease patients is low and further disturbed by the host’s inflammatory response and presence of obstructive jaundice. A number of studies have investigated additional biomarkers, but their individual diagnostic accuracy is seldom higher than that of CA19-9 [13]. Where it has recently been suggested that the combination of CA19-9 and an isoform of apolipoprotein A2 may be useful in the early detection of PDAC prior to diagnosis [14], improved differential diagnostic accuracy has also been reported for the ratio of the increase-folds of CA19-9 to that of total bilirubin in obstructive jaundice patients [15] and for combined biomarker panels including CA19-9 in discriminating PDAC patients from benign pancreatic diseases [13]. From our recent systematic review on the role of inflammatory cytokines and angiogenic factors as potential biomarkers in PDAC, six biomolecules stood out from the literature and included interleukin (IL)-1β, -6, -8, -10, transforming growth factor (TGF) and vascular endothelial growth factor (VEGF) [16]. These biomolecules, in addition to tumour necrosis factor (TNF)-α, are also secreted from the tumour microenvironment [17, 18]. Indeed, an angiogenic switch, or activation of angiogenesis from a quiescent state, is believed to occur during the initial stages of pancreatic cancer when the tumour cells are still dormant [19], despite hypoxia and the low microvascular density found in PDAC [9, 20–26].

Effective primary screening using circulating biomarker panels for the discrimination of PDAC patients from benign disease patients is a promising direction for aiding earlier diagnosis and improving prognosis, but few studies have investigated large arrays of circulating cytokines and angiogenic factors, and/or developed a diagnostic panel for accurate discrimination with good sensitivity and specificity [9, 13, 23]. We investigated whether combinations of inflammatory cytokines and angiogenic factors could facilitate the earlier diagnosis of PDAC in our South African setting.

## Materials and methods

### Ethical considerations

Ethical approval was obtained from the University of the Witwatersrand Human Research Ethics Committee (Medical) (clearance number M140669) and from the both the hospitals’ management of the Chris Hani Baragwanath Academic Hospital and the Charlotte Maxeke Johannesburg Academic Hospital. The study was conducted according to the Helsinki declaration (2008 amended version) and written informed consent was obtained from each patient preoperatively and from all control participants.

### Patient characteristics

The present prospective study follows a pilot investigation in Black South African patients of the potential of angiogenic factors and inflammatory cytokines as biomarkers in South African pancreatic ductal adenocarcinoma [9]. A genetically homogenous population group of 174 participants was recruited from the two public hospitals in Johannesburg, South Africa, and included 99 PDAC patients (diagnosed histologically or cytologically) and 75 ethnic-matched, benign control participants with abdominal CT scan demonstrating a normal pancreas and no history of any cancer type. The 75 benign control participants were further sub-grouped into the benign biliary pathology (BBP) group, including 29 patients with either obstructive jaundice, common bile duct injury, choledocholithiasis, or cholecystitis, and the non-hepato-pancreato-biliary (HPB) benign control (BC) group, including 46 patients with either abdominal aortic aneurysm, critical limb ischemia, or other abdominal pathology. Finally, PDAC in the presence of obstructive jaundice (head lesions) was compared to the subgroup of BBP patients with obstructive jaundice, choledocholithiasis or common bile duct injury (n = 27). The level of total bilirubin was used to reflect the degree of obstruction and the indication for stenting was determined by the extent of complications and injury, as per current practice.

### Data and blood sample collection

Data and sample collection was done as previously described in our pilot study [9]. Briefly, demographic and clinical data were recorded on standardized questionnaires and related to patient co-morbidities. In addition to standard laboratory blood tests, blood tests routinely requested by PDAC-treating clinicians, were recorded. Cytokine and angiogenic factor measurements were conducted in 75% of our study participants (n = 131/174), of which 85 were PDAC, 25 BBP and 21 BC patients. In total, 10 mL of whole blood was collected into ethylenediaminetetraacetic acid (EDTA) tubes from these 131 patients. Plasma was subsequently separated by centrifugation at 4°C for 10 min at 1000 x g, and stored in 500 μL aliquots at -80°C until analysed.

### Measurement of inflammatory cytokines and angiogenic biomarkers

Plasma levels of 38 inflammatory cytokines and angiogenic factors were measured blindly in duplicate at the second freeze-thaw cycle using commercially available human magnetic multiplex screening assays according to the manufacturer’s instructions. Specifically, the Bio-Plex Pro^™^ Human Cytokine 27-plex measured IL-1β, -1ra, -2, -4, -5, -6, -7, -8, -9, -10, -12p, -13, -15, 17A, Eotaxin, bFGF, G-CSF, GM-CSF, interferon (IFN)-γ, IFN-γ inducible protein (IP-10 or CXCL10), MCP-1, MIP-1α, -1β, PDGF-BB, RANTES, TNF-α and VEGF. The Bio-Plex Pro^™^ Human Th17 3-Plex measured IL-21, -22 and -23. The biomolecules acidic FGF, P-selectin, PDGF-AA, PlGF, VEGF-R1/Flt-1, VEGF R2/KDR and adhesion molecules (ICAM-1 and VCAM-1) were measured using customised R&D Systems Human magnetic luminex screening assays. The minimum detectable concentrations for the 27 inflammatory cytokines and 11 angiogenic factors in our laboratory are given in S1 Table.

### Statistical analyses

All statistical analyses were conducted using STATISTICA Version 13.2 and STATA Version 14.2 suites of analytics software. The Shapiro-Wilk W test was performed to determine the normality of data distribution. For the three inflammatory cytokines IL-2, -21 and -23, the majority of measurements were below the limit of detection (LOD) and these values (91%, 62% and 80%, respectively) were substituted by ½*LOD for statistical analyses. The same was done for the values below the LOD for IL-15 (29%), GM-CSF (<1%), VEGF (1.5%) and IL-22 (24%). Differences in clinical characteristics and biomarker levels between PDAC patients and benign controls were assessed using non-parametric Mann-Whitney U and Kruskal-Wallis tests. The biochemical, cytokine and angiogenic factor measurements are presented as raw median values and interquartile ranges (IQRs). The Pearson’s Chi-squared test and Fishers’ exact test, where appropriate, were conducted for analyses of categorical variables and the results are expressed as absolute and relative frequencies.

Univariate and multivariate logistical regression analyses were conducted on logarithmic transformed values for risk analysis and model building, and the Hosmer-Lemeshow Test was applied to determine the goodness-of-fit of the panels. All statistical tests were two-tailed and 5% level of significance was considered significant. Regression analyses on logarithmic transformed values were modelled against the probability that a participant has PDAC. Only variables with p-values < 0.2 in the univariate analyses were considered for multivariate model building. Receiver Operating Characteristic (ROC) curve analysis was conducted and area under the curve (AUC) reported to determine the ability of the individual biomarkers or biomarker panels to accurately diagnose PDAC.

## Results

### Demographic and clinical characteristics of the study population

The demographic and clinical characteristics of the study population are summarised in Table 1. PDAC patients had a mean (±SD) age of 60.0 (±11.3) years and were age-matched with the BC group [60.2 (±12.1); p = not significant (NS)], yet significantly older than the BBP group [47.5 (±12.6); p < 0.0001]. The prevalence of diabetes was 23.7%, 11.1% and 17.4% in the PDAC, BBP and BC patients, respectively. When comparing PDAC to BC in Table 1 we observed significant gender differences and significantly higher levels (all p-values <0.0001) for the following biochemical measures, albeit some within normal ranges: gamma-glutamyl transferase (GGT), total bilirubin, carbohydrate or cancer antigen 19–9 (CA19-9), and carcinoembryonic antigen (CEA).

**Table 1:** Demographic and clinical characteristics of PDAC patients and controls.

Parameters	All	PDAC	BBP	BC	P-value*		
	(n = 174)	(n = 99)	(n = 29)	(n = 46)	PDAC vs BBP+BC	PDAC vs BBP	PDAC vs BC
Age, years (mean ± SD)	58.0 ± 12.6	60.0 ± 11.3	47.5 ± 12.6	60.2 ± 12.1	**0.02**	**<0.0001**	NS
Gender, n (%)							
*Male*	94 (54.0%)	54 (54.5%)	7 (24.1%)	33 (71.7%)	NS	**0.003**	0.04
Smoking status, n (%)	*n = 168*	*n = 93*	*n = 29*	*n = 46*			
*Current/quit smoker*	97 (57.7%)	55 (59.1%)	9 (31.0%)	33 (71.7%)	NS	**0.007**	NS
*Never smoked*	71 (42.3%)	38 (40.9%)	20 (69.0%)	13 (28.3%)			
Alcohol, n (%)	*n = 167*	*n = 92*	*n = 29*	*n = 46*			
*Yes*	101 (60.5%)	64 (69.6%)	11 (37.9%)	26 (56.5%)	**0.006**	**0.002**	NS
BMI, kg/m^2^ (mean ± SD)	24.0 ± 7.7	23.3 ± 7.6	28.9 ± 8.3	21.9 ± 5.3	NS	**0.006**	NS
*Biochemical measures*							
GGT (units/L)	313.0 (83.0–656.0)	450.0 (149.0–797.0)	357.0 (103.0–564.0)	60 (34.0–123.0)	**<0.0001**	NS	**<0.0001**
Total bilirubin (μmol/L)	48.0 (7.0–209.0)	125.0 (29.0–312.0)	48.0 (9.0–115.0)	6.5 (5.0–9.0)	**<0.0001**	**0.009**	**<0.0001**
HbA1c (%)	5.9 (5.3–7.2)	6.1 (5.1–7.3)	5.3 (4.6–6.5)	5.9 (5.5–6.9)	NS	NS	NS
Platelet count (10^9^/L)	337.5 (252.0–470.0)	323.5 (252.0–427.0)	366.0 (246.0–501.0)	375.0 (264.0–473.0)	NS	NS	NS
CRP (mg/L)	53.0 (18.0–116.0)	52.0 (18.0–116.0)	51.0 (13.0–99.0)	60.0 (25.0–116.0)	NS	NS	NS
CA19-9 (U/mL)	76.0 (21.4–1884)	327.0 (32.0–5660)	76.0 (8.0–230.0)	23.0 (20.4–26.0)	**<0.0001**	NS	**<0.0001**
CEA (ng/mL)	3.9 (2.2–9.8)	6.2 (2.9–14.1)	2.0 (1.8–3.1)	2.2 (1.6–2.5)	**<0.0001**	**0.01**	**<0.0001**
CA19-9/Total bilirubin	4.8 (0.4–24.7)	5.9 (0.3–48.2)	1.3 (0.3–4.7)	3.7 (1.4–6.5)	NS	NS	NS

*Mann-Whitney test for continuous variables, Pearson chi2^2^ or Fisher’s exact test for categorical variables. Continuous variables are presented as median (interquartile range [IQR], unless specified otherwise. Categorical variables are expressed as absolute and relative frequencies.

*Abbreviations*: BBP, benign biliary pathology; BC, benign controls; BMI, body mass index; CA19-9, carbohydrate or cancer antigen 19–9; CEA, carcinoembryonic antigen; CRP, C-reactive protein; GGT, gamma-glutamyl transferase; HbA1c, glycated hemoglobin A1c; NS, non-significant; PDAC, pancreatic ductal adenocarcinoma; SD, standard deviation.

When comparing PDAC to BBP, we found statistically significant differences for gender, smoking and alcohol exposures, BMI, total bilirubin and CEA, and no difference for CA19-9 levels.

### Circulating inflammatory cytokines and angiogenic markers in PDAC

Circulating inflammatory cytokines (Table 2) and angiogenic biomolecule levels (Table 3) in PDAC were compared to BC and BBP participants. Of the 38 measured potential biomarkers, 12 biomolecules and two of their ratios showed statistically significant differences between the PDAC and BC group. Where IL-8 (p < 0.001), CXCL10 (p < 0.0001), MIP-1β (p < 0.003) and the VEGF-R1/VEGF-R2 ratio (p < 0.0001) were significantly elevated in PDAC, GM-CSF (p = 0.007), IFN-γ (p = 0.007), IL-4 (p = 0.04), IL-5 (p = 0.008), IL-15 (p = 0.001), IL-17 (p = 0.02), MIP-1α (p = 0.05), bFGF (p = 0.02) and the VEGF/sVEGF-R1 ratio (p = 0.02) were significantly decreased.

**Table 2:** Circulating levels of inflammatory cytokines and chemokines in PDAC patients and control participants.

Cytokines	All	PDAC patients	BBP	BC	P-value*		
(pg/mL)	(n = 131)	(n = 85)	(n = 25)	(n = 21)	PDAC vs BBP+BC	PDAC vs BBP	PDAC vs BC
Eotaxin	105.7 (84.4–132.7)	109.3 (87.9–136.8)	96.1 (83.7–109.5)	104.2 (84.7–138.0)	NS	**0.04**	NS
G-CSF	204.8 (152.9–258.4)	203.8 (147.5–258.4)	228.0 (173.5–259.8)	192.1 (160.2–251.1)	NS	NS	NS
GM-CSF	41.9 (27.1–59.2)	36.4 (21.4–55.5)	42.2 (28.5–56.1)	53.5 (40.0–92.0)	**0.02**	NS	**0.007**
IFN-γ	265.9 (191.5–346.8)	235.5 (179.4–318.8)	313.2 (259.5–334.9)	363.4 (263.1–429.8)	**0.0006**	**0.01**	**0.007**
IL-1β	7.8 (6.1–10.2)	7.3 (5.4–9.8)	8.5 (6.8–9.8)	9.7 (6.9–13.6)	0.06	NS	0.07
IL-1ra	345.9 (255.4–439.9)	351.9 (255.2–455.4)	321.2 (270.0–399.7)	357.2 (255.4–443.2)	NS	NS	NS
IL-2†	0.125 (0.125–0.13)	0.125 (0.125–0.13)	0.125 (0.125–0.13)	0.125 (0.125–0.125)	NS	NS	NS
IL-4	9.3 (7.6–11.6)	8.5 (6.8–11.4)	10.7 (9.3–12.1)	10.5 (9.3–12.7)	**<0.001**	**0.003**	**0.04**
IL-5	32.4 (23.5–42.3)	30.3 (21.1–39.9)	35.3 (30.8–41.8)	38.7 (35.0–53.2)	**0.002**	**0.04**	**0.008**
IL-6	30.05 (20.0–52.3)	31.7 (20.8–53.8)	26.6 (17.4–31.7)	31.7 (24.5–49.1)	NS	NS	NS
IL-7	35.7 (26.8–42.2)	34.6 (22.2–41.9)	35.3 (30.5–42.1)	36.8 (34.3–44.4)	NS	NS	NS
IL-8	66.6 (46.9–104.8)	75.2 (49.3–115.7)	52.2 (47.2–75.3)	48.0 (39.6–67.4)	**0.0004**	**0.03**	**0.0009**
IL-9	87.3 (65.0–108.6)	87.2 (62.6–108.6)	79.6 (69.0–93.1)	95.7 (85.1–114.3)	NS	NS	NS
IL-10	34.0 (22.9–50.3)	31.7 (21.8–47.2)	34.8 (26.4–43.2)	43.3 (24.2–68.5)	NS	NS	NS
IL-12	48.6 (35.0–77.4)	45.9 (33.5–72.0)	48.7 (41.0–75.2)	67.3 (39.1–115.6)	NS	NS	NS
IL-13	19.1 (13.0–26.3)	19.8 (11.8–26.6)	18.8 (15.6–27.1)	18.8 (13.0–23.9)	NS	NS	NS
IL-15	22.3 (0.73–41.2)	13.7 (0.73–32.5)	33.0 (17.8–43.2)	36.0 (23.1–52.6)	**0.0001**	**0.004**	**0.001**
IL-17	397.7 (268.9–524.8)	354.0 (237.9–476.5)	384.6 (326.5–483.7)	485.7 (316.8–658.3)	**0.03**	NS	**0.02**
IL-21†	0.10 (0.10–18.0)	0.10 (0.10–22.0)	0.10 (0.10–23.3)	0.10 (0.10–17.0)	NS	NS	NS
IL-22	3.4 (0.2–8.5)	3.7 (0.8–8.9)	1.1 (0.1–10.7)	4.0 (0.1–7.4)	NS	NS	NS
IL-23†	3.01 (3.01–3.01)	3.01 (3.01–3.01)	3.01 (3.01–3.01)	3.01 (3.01–3.01)	NS	NS	NS
CXCL10 (IP-10)	1246 (534–2285)	1440 (839.2–2870)	1002 (618.9–1514)	472.1 (411.7–607.5)	**<0.0001**	**0.04**	**<0.0001**
MCP-1	105.2 (78.9–130.9)	98.6 (78.1–132.2)	107.2 (102.8–126.9)	102.4 (90.8–130.9)	NS	NS	NS
MIP-1α (CCL3)	8.5 (6.9–11.2)	8.1 (6.7–10.7)	8.7 (7.5–10.5)	10.6 (7.6–14.3)	NS	NS	0.05
MIP-1β (CCL4)	110.3 (72.4–162.9)	125.4 (81.4–183.8)	100.6 (73.1–126.6)	71.4 (51.2–92.7)	**0.0003**	**0.05**	**0.0003**
RANTES (CCL5)	8906 (6845–11820)	8985 (6659–11686)	9901 (8019–11139)	8183 (6845–11820)	NS	NS	NS
TNF-α	140.5 (109.4–171.4)	138.7 (102.8–171.4)	137.4 (119.4–157.7)	152.6 (130.4–197.7)	NS	NS	NS

*Mann-Whitney test statistic. Values presented as median (interquartile range [IQR]).

^†^Majority of these values were below the limit of detection (LOD) and substituted with ½*LOD; excluding these values from the analysis (data not shown) did not give significant differences between the groups.

*Abbreviations*: BBP, benign biliary pathology; BC, benign controls; CCL, chemokine ligand; G-CSF, granulocyte colony-stimulating; GM-CSF, granulocyte-macrophage colony-stimulating; IFN, interferon; IL, interleukin; IP, interferon γ-induced protein; MCP-1, monocyte chemoattractant protein; MIP, macrophage inflammatory protein; NS, non-significant; PDAC, pancreatic ductal adenocarcinoma; RANTES, regulated on activation, normal T cell expressed and secreted; TNF, tumour necrosis factor.

**Table 3:** Circulating levels of angiogenic factors in PDAC patients and control participants.

Factor	All	PDAC patients	BBP	BC	P-value*		
	(n = 131)	(n = 85)	(n = 25)	(n = 21)	PDAC vs BBP+BC	PDAC vs BBP	PDAC vs BC
aFGF (pg/mL)	10.2 (7.0–17.8)	10.1 (6.4–17.3)	7.8 (6.2–13.9)	10.3 (9.1–18.3)	NS	NS	NS
bFGF (pg/mL)	114.5 (91.4–144.6)	110.5 (87.3–136.7)	117.1 (97.5–139.6)	130.7 (101.4–179.2)	**0.04**	NS	**0.02**
ICAM-1(ng/mL)	174.6 (743.7–413.8)	195.8 (80.8–416.8)	131.4 (76.8–219.7)	167.2 (74.2–584.5)	NS	NS	NS
VCAM-1(ng/mL)	1806 (1225–2335)	1913 (1507–2611)	2984 (1055–6540)	1593 (1272–2047)	NS	NS	NS
P-selectin (ng/mL)	38.1 (26.9–51.5)	36.5 (30.3–41.1)	49.2 (27.0–75.4)	41.1 (25.0–51.5)	NS	NS	NS
PDGF-AA (pg/mL)	923.9 (485.1–1563)	796.5 (458.5–1360)	1315 (828.0–1848)	1007 (502.4–1563)	0.06	**0.04**	NS
PDGF-BB (pg/mL)	1531 (946–2459)	1520 (839–2374)	1359 (1024–2064)	2196 (1193–2914)	NS	NS	NS
PlGF (pg/mL)	9.0 (2.3–21.5)	5.9 (1.9–18.9)	14.3 (5.7–42.8)	9.1 (2.4–19.7)	0.07	**0.03**	NS
VEGF (pg/mL)	91.5 (60.3–168.9)	87.8 (60.3–155.9)	94.9 (59.9–147.6)	126.7 (74.3–207.5)	NS	NS	NS
sVEGF-R2/KDR	7883 (6040–11509)	7814 (5785–10900)	7845 (6605–12457)	8867 (6756–11756)	NS	NS	NS
sVEGF-R1/Flt-1	140.7 (82.9–222.7)	155.7 (104.6–246.7)	141.0 (92.3–196.4)	83.0 (60.6–140.7)	**0.01**	NS	**0.001**
VEGF/sVEGF-R1	0.65 (0.34–1.76)	0.61 (0.27–1.44)	0.64 (0.35–1.23)	1.55 (0.53–4.71)	NS	NS	**0.02**
VEGF/sVEGF-R2	0.013 (0.007–0.026)	0.013 (0.007–0.027)	0.011 (0.008–0.024)	0.017 (0.007–0.026)	NS	NS	NS
sVEGF-R1/-R2	0.017 (0.010–0.031)	0.022 (0.014–0.036)	0.015 (0.010–0.031)	0.011 (0.007–0.015)	**0.001**	NS	**<0.0001**

*Mann-Whitney test statistic. Values presented as median (interquartile range [IQR]).

*Abbreviations*: BBP, benign biliary pathology; BC, benign controls; FGF, fibroblast growth factor; aFGF, acidic FGF; bFGF, basic FGF; ICAM-1, intercellular cell adhesion molecule-1; NS, non-significant; PDAC, pancreatic ductal adenocarcinoma; PDGF, platelet-derived growth factor; PlGF, placental growth factor; VCAM-1, vascular cell-adhesion molecule-1; VEGF, vascular endothelial growth factor; sVEGF-R, soluble form of vascular endothelial growth factor receptor.

Ten of the measured biomolecules were statistically significantly altered between PDAC and BBP: plasma levels of Eotaxin (p = 0.04), IL-8 (p = 0.03), CXCL10 (p = 0.04) and MIP-1β (p = 0.05) were increased in the PDAC group, whereas IFN-γ (p = 0.01), IL-4 (p = 0.003), IL-5 (p = 0.04), IL-15 (p = 0.004), PDGF-AA (p = 0.04) and PlGF (p = 0.03) were significantly decreased.

### Clinical characteristics and circulating biomarkers in PDAC patients with obstructive jaundice

Table 4 shows the level of total bilirubin per subgroup of patients which reflects the increased degree of obstruction in PDAC patients compared to benign jaundiced patients. Table 5 shows the demographical and clinical characteristics, as well as the circulating biomarkers that differ significantly in the subgroup analysis of PDAC patients in the presence of jaundice when compared to BBP patients with either obstructive jaundice, choledocholithiasis or common bile duct injury. Notably, only marginally significant differences were detected between the median levels of CA19-9 (p = 0.05). Age differences were extremely significant (p < 0.0001) and smoking and alcohol exposure were significantly higher in the PDAC group. Even though the average BMI of PDAC patients compared to the overweight BMI in benign patients reached statistical significance, this may not be clinically important as BMI may reduce in the period leading up to a PDAC diagnosis. Total bilirubin, CEA and IL-8 levels were significantly raised in jaundiced PDAC, whist IL-4, IL-15 and PlGF were significantly decreased when compared to BBP controls.

**Table 4:** Total bilirubin levels in patients with PDAC (with or without obstructive jaundice) and benign disease in the presence of jaundice, choledocholithiasis or common bile duct injury.

Patient subgroups	Total Bilirubin (μmol/L)	P value
**PDAC**	125.0 (29.0–312.0)	
Obstructive jaundice (n = 52)	200.5 (38.0–365.0)	**0.01**
Without jaundice (n = 40)	74.5 (7.0–245.0)	
**BBP**	49.0 (18.0–115.0)	
Jaundice (n = 5)	113.0 (110.0–141.0)	NS
Choledocholithiasis (n = 20)	44.5 (9.0–115.0)	
Common bile duct injury (n = 2)	39.0 (30.0–48.0)	

Data are presented as median (interquartile range [IQR].

**Table 5:** Demographics, clinical characteristics and circulating biomarkers of patients with PDAC in the presence of jaundice vs benign disease in the presence of jaundice, choledocholithiasis or common bile duct injury.

Parameters*	PDAC—Jaundice	BBP	P-value
	(n = 52)	(n = 27)	
Age, years (mean ± SD)	60.5 ± 11.4	46.9 ± 12.7	**<0.0001**
Male Gender, n (%)	29 (55.8%)	7 (25.9%)	**0.01**
Smoking exposure, n (%)	32 (61.5%)	9 (33.3%)	**0.02**
Alcohol exposure, n (%)	38 (73.1%)	10 (37.0%)	**0.002**
BMI, kg/m^2^ (mean ± SD)	22.9 ± 6.2	29.2 ± 8.3	**0.004**
*Biochemical measures*			
Total bilirubin (μmol/L)	200.5 (38.0–365.0)	49.0 (18.0–115.0)	**0.001**
HbA1c (%)	5.9 (5.1–7.6)	5.1 (4.4–5.8)	0.05
CA19-9 (U/mL)	715.7 (49.0–3863)	76.0 (8.0–230.0)	0.06
CEA (ng/mL)	5.3 (3.0–12.5)	2.0 (1.8–3.1)	**0.01**
*Cytokines & angiogenic factors*	(*n = 48)*	(*n = 25)*	
IFN-γ (pg/mL)	234.4 (179.4–329.2)	313.2 (259.5–334.9)	**0.04**
IL-4 (pg/mL)	8.8 (7.1–11.4)	10.7 (9.3–12.1)	**0.01**
IL-8 (pg/mL)	84.6 (58.3–121.4)	52.2 (47.2–75.3)	**0.02**
IL-15 (pg/mL)	13.6 (0.73–41.2)	33.0 (17.8–43.2)	**0.02**
PlGF (pg/mL)	4.2 (1.6–14.6)	14.3 (5.7–42.8)	**0.01**

*Only significant parameters shown. Continuous variables are presented as median (interquartile range [IQR], unless specified otherwise. Categorical variables expressed as absolute and relative frequencies. *Abbreviations*: BMI, body mass index; CA19-9, carbohydrate or cancer antigen 19–9; CEA, carcinoembryonic antigen; HbA1c, glycated hemoglobin A1c; PDAC, pancreatic ductal adenocarcinoma; SD, standard deviation.

### Classification modelling to distinguish PDAC patients from benign control (BC) participants

**Fig 1:**
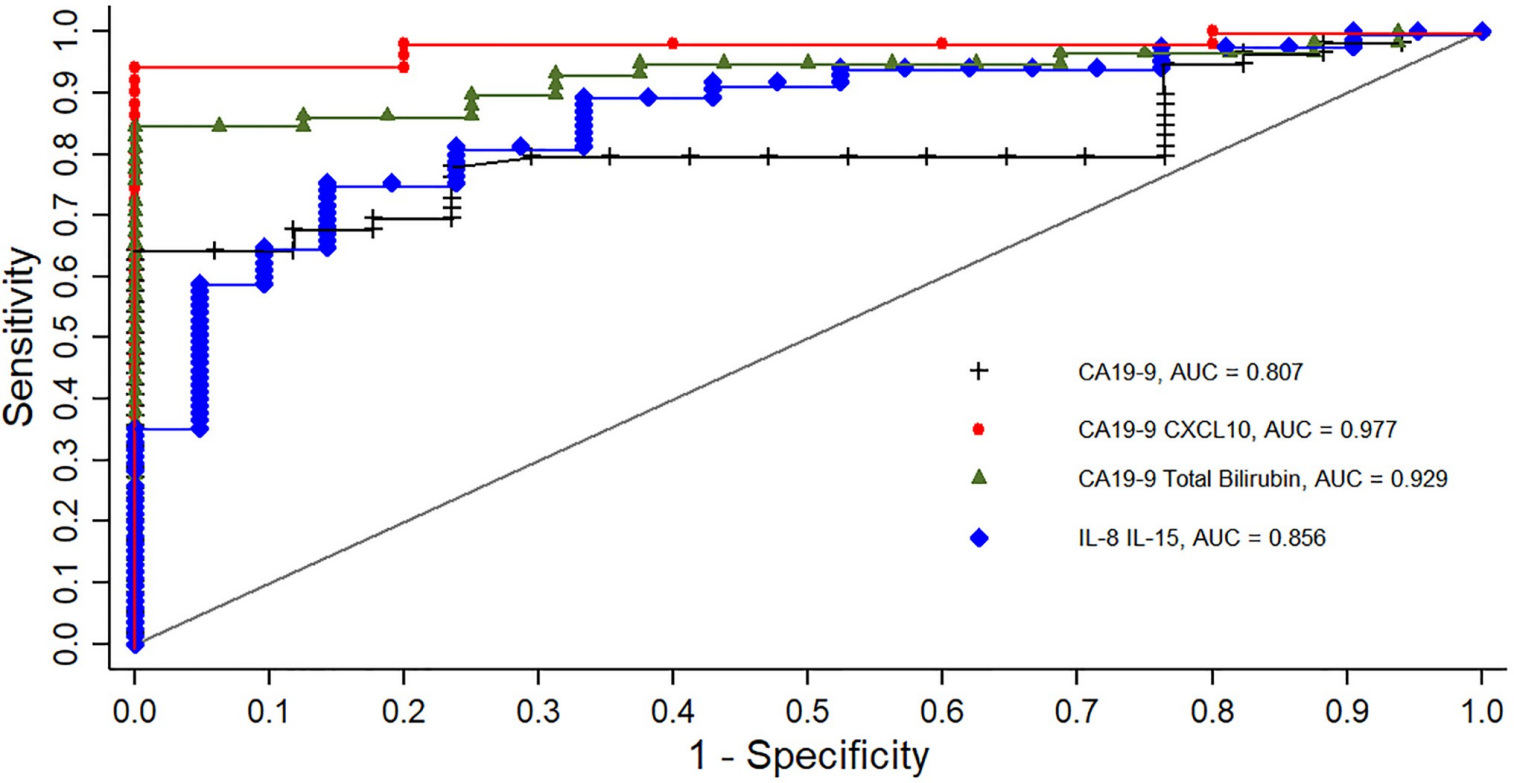
Comparison of ROC curves for combined panels vs CA19-9 in distinguishing PDAC from BC.

Univariate logistic regression analysis of our routine clinical and biochemical measures, as well as the measured circulating cytokines and angiogenic markers, showed that the following variables significantly discriminated PDAC patients from BC participants: Male gender, GGT, total bilirubin, CA19-9, CEA, GM-CSF, IFN-γ, IL-8, IL-15, CXCL10 (IP-10), MIP-1β, bFGF, sVEGF-R1, VEGF/sVEGF-R1 ratio and sVEGF-R1/sVEGF-R2 ratio. These variables, along with those variables with p-values less than 0.2 in the univariate analysis (results not shown), were considered in multivariate logistic regression model building. Table 6 shows univariate results of biomarkers that contributed significantly to our multivariate models, as well as these best-fit multivariate panels. As a combined panel, CA19-9 and CXCL10 (Panel 1) demonstrated improved diagnostic power over CA19-9 alone in our study population (AUC = 0.977 vs AUC = 0.807, respectively; p = 0.001; Table 6 and Fig 1). This best-fit panel is evidenced by low Hosmer-Lemeshow (H-L) test statistic and high H-L p-value approximating 1. Similarly, a panel comprising CA19-9 and total bilirubin (Panel 2) achieved a greater AUC compared to CA19-9 alone (AUC = 0.929 vs AUC = 0.807, respectively; p = 0.02). A third panel modelled with IL-8 and IL-15 whilst excluding CA19-9 (Panel 3; AUC = 0.856), also demonstrated diagnostic power in an improved good-fit model, although this AUC was not significantly higher than that of CA19-9 alone (p = 0.22).

**Table 6:** Univariate^a^ biomarkers and multivariate classification panels to distinguish PDAC from BC.

Effect	AUC	AUC 95% CI	Odds Ratio	OR 95% CI	P-value	HL-chi^2^	HL P-value
CA19-9	0.807	0.710–0.903	3.47	1.62–7.41	0.001	26.9	<0.001
CXCL10 (IP-10)	0.801	0.699–0.902	21.7	4.2–112.0	<0.0001	8.0	0.44
IL-8	0.736	0.623–0.849	60.5	4.4–838.3	0.002	6.9	0.55
IL-15	0.723	0.607–0.839	0.29	0.12–0.68	0.004	4.7	0.45
Total bilirubin	0.854	0.789–0.919	9.56	4.1–22.5	<0.0001	4.7	0.79
***PDAC vs BC panel 1***	**0.977**	**0.939–1.000**			**<0.0001**	0.27	~1
CA19-9			24.8	1.20–516.3	0.04		
CXCL10 (IP-10)			1,399	1.89–1,037e+03	0.03		
***PDAC vs BC panel 2***	**0.929**	**0.872–0.986**			**<0.0001**	4.7	0.79
CA19-9			4.10	1.34–12.6	0.01		
Total bilirubin			16.2	2.77–94.2	0.002		
***PDAC vs BC panel 3***	**0.856**	**0.773–0.939**			**<0.0001**	5.8	0.67
IL-8			373.0	11.7–11,931	0.001		
IL-15			0.16	0.05–0.50	0.001		

^a^ Modelled probability that participant has PDAC; only significant variables contributing to panels listed. Statistics were conducted on log transformed values of variables. *Abbreviations*: CA19-9, carbohydrate or cancer antigen 19–9; CEA, carcinoembryonic antigen; HL, Hosmer-Lemeshow test; IL, interleukin; IP, interferon γ-induced protein; OR, odds ratio.

### Classification modelling to distinguish PDAC patients from benign biliary pathology participants

**Fig 2:**
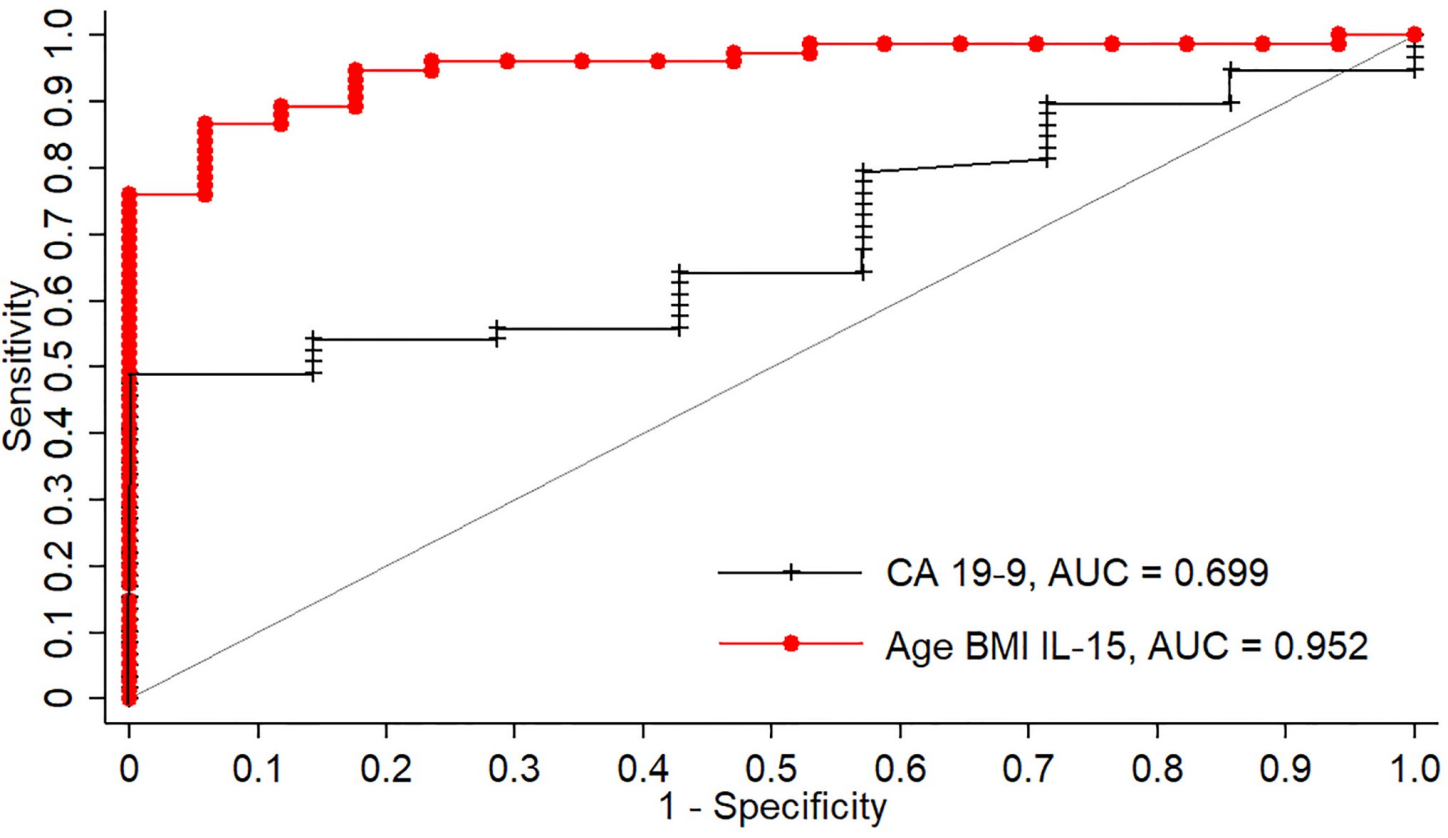
Comparison of ROC curve for combined panel vs CA19-9 in distinguishing PDAC from BBP.

Univariate logistic regression analysis of our variables showed that the following variables significantly discriminated PDAC patients from BBP participants: Age, male gender, smoking exposure, alcohol consumption, BMI, total bilirubin, Eotaxin, IFN-γ, IL-4, IL-8, IL-15 and PDGF-AA. These variables, along with those variables with p-values less than 0.2 in the univariate analysis (results not shown), were considered in multivariate logistic regression model building. Table 7 shows univariate results from biomarkers considered in multivariate models and these best-fit multivariate panels. Where CA19-9 alone could not discriminate between PDAC and BBP in our study population (p = 0.10), a combined panel including age, BMI and IL-15 showed great diagnostic power in our study population (AUC = 0.952, p < 0.0001; Table 7 and Fig 2). Clinically, BMI may cause concern in a biomarker panel as the patient’s weight may decrease significantly in the period leading up to a PDAC diagnosis. Removing BMI from our panel still yields a significantly, good-fit model (AUC = 0.872, p < 0.0001), albeit at a reduced AUC. The AUCs of these two panels differ with borderline significance (p = 0.05).

**Table 7:** Univariate^a^ biomarkers and multivariate classification panels to distinguish PDAC from BBP.

Effect	AUC	AUC 95% CI	Odds Ratio	OR 95% CI	P-value	HL-chi^2^	HL P-value
Age	0.798	0.695–0.900	1.10	1.05–1.15	<0.0001	17.1	0.03
BMI	0.707	0.577–0.837	0.92	0.86–0.98	0.007	8.6	0.38
IL-15	0.685	0.580–0.791	0.36	0.18–0.74	0.005	9.3	0.10
CA19-9	0.699	0.536–0.861	1.86	0.90–3.86	0.096	6.6	0.58
***PDAC vs BBP panel 1***	**0.952**	**0.909–0.995**			**<0.0001**	3.2	0.92
Age			1.17	1.08–1.27	<0.0001		
BMI			0.86	0.77–0.95	0.003		
IL-15			0.18	0.05–0.72	0.02		
***PDAC vs BBP panel 2***	**0.872**	**0.785–0.959**			**<0.0001**	9.8	0.28
Age			1.13	1.07–1.20	<0.0001		
IL-15			0.35	0.15–0.81	0.01		

^a^ Modelled probability that participant has PDAC; only significant variables contributing to panels listed. Statistics were conducted on log transformed values of variables, with the exception of age and BMI. *Abbreviations*: BMI, body mass index; HL, Hosmer-Lemeshow test; IL, interleukin; OR, odds ratio.

### Classification modelling to distinguish PDAC in the presence of jaundice from benign disease in the presence of jaundice, choledocholithiasis or common bile duct injury

**Fig 3:**
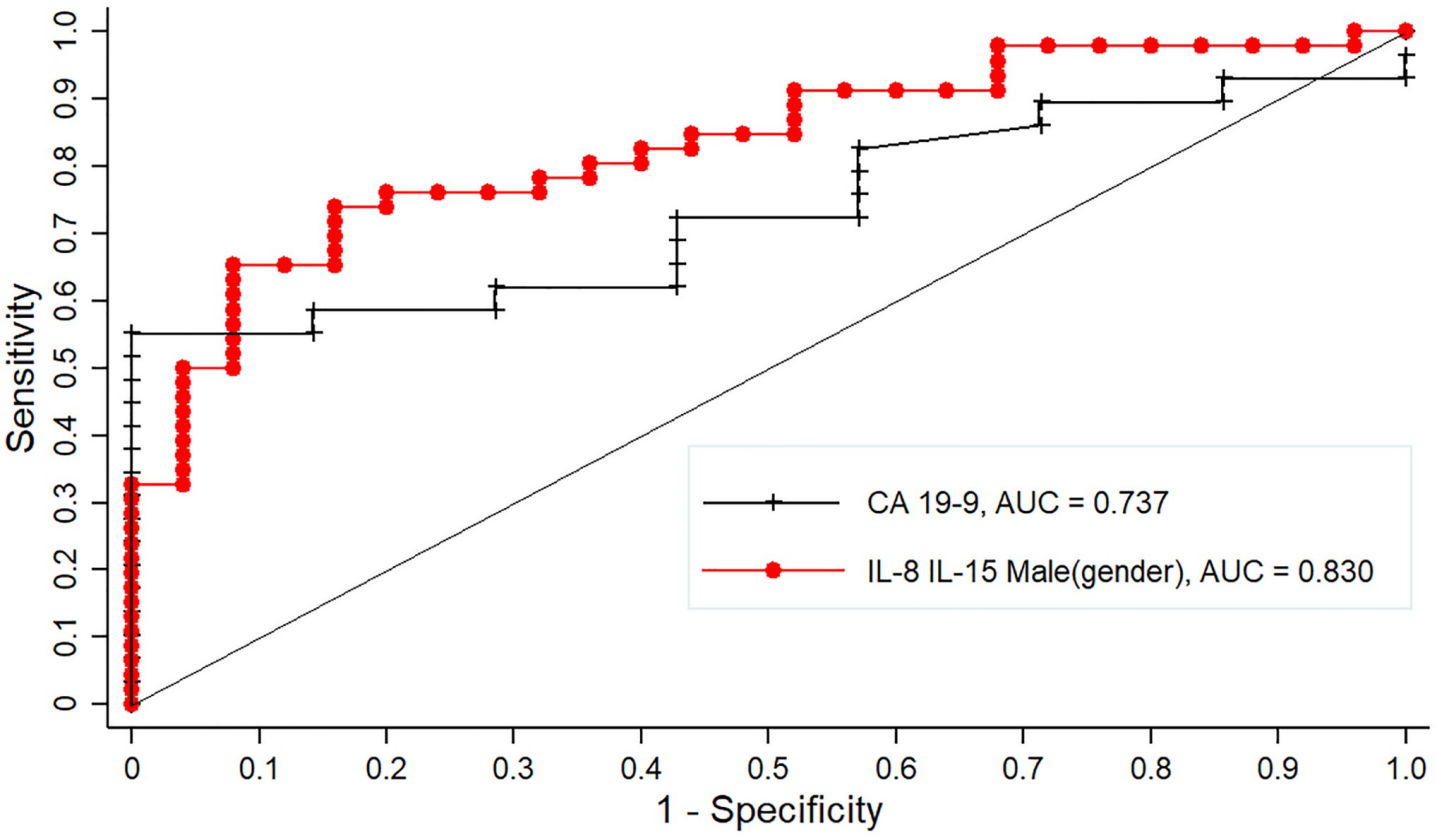
Comparison of ROC curves for a combined panel vs CA19-9 in distinguishing PDAC in the presence of jaundice from benign patients in the presence of jaundice, choledocholithiasis or common bile duct injury.

Of all PDAC patients in our study, 56.5% presented with obstructive jaundice. Univariate logistic regression identified variables that significantly distinguish PDAC in the presence of jaundice from the benign subgroup of patients with either obstructive jaundice (n = 5), choledocholithiasis (n = 20) or common bile duct injury (n = 2). These variables included age (p < 0.0001), male gender (p = 0.02), smoking exposure (p = 0.03), history of alcohol consumption (p = 0.004), BMI (p = 0.007), total bilirubin (p = 0.007), IL-8 (p = 0.02) and IL-15 (0.01). Again, CA19-9 alone could not discriminate between PDAC in the presence of jaundice and BBP (p = 0.06). The best-fit multivariate model from these biomarkers was a diagnostic panel of IL-8, IL-15 and male gender (p < 0.0001; Table 8 and Fig 3).

**Table 8:** Univariate^a^ biomarkers and multivariate classification panels to distinguish PDAC in the presence of jaundice from benign disease in the presence of jaundice, choledocholithiasis or common bile duct injury.

Effect	AUC	AUC 95% CI	Odds Ratio	OR 95% CI	P-value	HL-chi^2^	HL P-value
CA19-9	0.737	0.567–0.906	2.24	0.97–5.18	0.06	8.4	0.39
IL-8	0.676	0.546–0.805	19.0	1.86–193.9	0.01	11.7	0.17
IL-15	0.670	0.546–0.795	0.37	0.174–0.770	0.01	14.4	0.03
Male gender	0.649	0.541–0.758	3.60	1.30–10.0	0.01	-	
***PDAC in OJ vs benign***	**0.830**	**0.735–0.925**			**<0.0001**	2.0	0.98
IL-8			45.5	2.46–842.9	0.01		
IL-15			0.24	0.09–0.63	0.004		
Male gender			4.99	1.38–18.1	0.01		

^a^ Modelled probability that participant has PDAC; only significant variables contributing to panels listed. Statistics were conducted on log transformed values of variables, with the exception of age and BMI. *Abbreviations*: BMI, body mass index; HL, Hosmer-Lemeshow test; IL, interleukin; OR, odds ratio.

Furthermore, in a separate analysis, the biomarker panel of IL-8, IL-15 and male gender remained significant in discriminating PDAC patients without jaundice from BBP patients (p < 0.001; data not shown), thereby ascertaining that these biomarkers were not directly related to the biliary obstruction.

## Discussion

Little improvement has been achieved in the survival rate of PDAC patients in recent decades, with the five-year survival rate at approximately 5% and surgical resection only an option in a limited number of eligible patients. Earlier diagnosis of PDAC is paramount and circulating biomarker panels of circulating biomolecules shows promise.

Findings from recent cytokine and angiogenic studies support the potential role for biomarker panels in the discrimination of PDAC patients from benign disease or healthy controls [9, 13, 23, 27]. Where CA19-9 is the established tumour marker for PDAC, its individual power to accurately distinguish PDAC from benign patients is low and disturbed by the host’s inflammatory response. From other biomarkers studies, individual cytokines or angiogenic factors fair no better than CA19-9 in accurately diagnosing PDAC from benign disease or healthy controls [13, 27]. Only a few studies have used combined biomarker panels, with or without CA19-9, in discriminating PDAC patients from benign control groups.

Consistent with other recent cytokine studies, [13, 16, 28, 29], we report significantly elevated IL-8 and CXCL10 values in our PDAC patients compared to benign control patients, with or without biliary pathology. As a potent pro-inflammatory cytokine, IL-8 expression in tumor cells and the tumor microenvironment plays a critical role in promoting tumoral angiogenesis and invasion of pancreatic cancer cells, as well as being a promising marker in the prognosis of PDAC [18, 30–32]. CXCL10, originally identified as a pro-inflammatory chemokine, critically exacerbates inflammation by increasing leukocyte homing into inflamed tissues, causing significant tissue damage [33]. The emerging role of CXCL10 in the pathogenesis of cancer has also included the inflammation-driven cancer, PDAC [13, 29]. Contrary to exhibiting anti-tumour actions and attenuating angiogenesis, CXCL10 also has tumour-promoting ability and has been associated with advanced human cancers, in particularly in breast cancer progression and metastasis [34–37], but also in PDAC [13, 29]. It is hypothesized that that pancreatic cancer cells induce stromal expression of CXCL10 in PDAC that contributes to an immunosuppressive and tumour-promoting microenvironment [29]. Both IL-8 and CXCL10 were significantly associated with having PDAC in our population, and CXCL10 in particular contributed significantly to a combined biomarker panel in enhancing diagnostic accuracy in PDAC from BC patients.

The pro-inflammatory cytokine IL-15 was protective against PDAC in our study population and also significantly contributed to our combined, diagnostic biomarker panels in distinguishing PDAC from BC and BBP patients. Moreover, IL-15 levels was protective against PDAC in patients with obstructive jaundice or choledocholithiasis. IL-15 binds to its receptor complex to activate an enhanced anti-tumour response that primarily stimulates the proliferation, activation and cytotoxic functions of natural killer (NK) and CD8 T cells, without stimulating immune-suppressing regulatory T cells [38, 39]. The American National Cancer Institute has identified IL-15 as one of the most promising immunotherapy targets for cancer [40] and the anti-tumour response of IL-15 has been well documented in experimental systems and in clinical cancer studies, notably in cutaneous T-cell lymphoma, leukemia of large granular lymphocytes and multiple myeloma [39]. Likewise, the beneficial effect of IL-15 in PDAC is also receiving increasing attention. Recently, Van Audenaerde et al. (2017) showed that IL-15 stimulated the NK cell-mediated death of pancreatic cancer and stellate cells [10]. Therefore, our results of lower IL-15 levels associated with a diagnosis of PDAC confirm the findings in these reports.

We performed multivariate logistic regression to ascertain the effects of various combined biomarker panels on the likelihood that a patient has PDAC. A best-fit panel of CA19-9 and CXCL10 displayed a significantly high probability of predicting PDAC from BC participants over and above CA19-9 alone (AUC = 0.977 vs. 0.807, p = 0.001). Moreover, assuming the other biomarker in the panel remains constant, the odds of having PDAC in our population is 24.8 times more likely with increasing CA19-9 values and at least 90% more likely with increasing CXCL10 concentrations. In another combined panel with CA19-9, total bilirubin levels significantly enhanced the diagnostic accuracy of predicting PDAC from BC compared to CA19-9 alone (AUC = 0.929 vs. 0.807, p = 0.02). In this latter panel, the odds of having PDAC is 4.1 times more likely with increasing CA19-9 and 16.2 times more likely with increasing total bilirubin levels.

The combined, best-fit panel consisting age, BMI and IL-15 showed significant diagnostic accuracy in predicting PDAC from BBP participants (AUC = 0.952, CI = 0.909–0.955; p < 0.0001). Assuming the biomarkers in the panel remain constant, the odds of having PDAC vs BBP is at least 8% more likely with each increasing year of age, at least 5% less likely with each increasing unit of BMI and at least 28% less likely with increasing IL-15 levels. Clinically, BMI may cause concern in a biomarker panel as the patient’s weight may decrease significantly in the period leading up to a PDAC diagnosis. Nevertheless, our biomarker panel remained powerful even after BMI was excluded (AUC = 0.872, CI = 0.785–0.959; p < 0.0001) and the likelihood of having PDAC increased by at least 7% for each year of advancing age and decreased by at least 19% with each unit increase in IL-15.

In patients with obstructive jaundice or choledocholithiasis, a panel of IL-8, IL-15 and gender significantly discriminated PDAC from benign disease with an AUC of 0.830 (p < 0.0001). Specifically, assuming the other biomarkers remain constant in the panel, the odds of having PDAC in this group of patients was 45.5 times more likely with increasing IL-8 concentrations, at least 37% less likely with increasing IL-15 concentrations and five times more likely in males than in females.

Notably, results from this study in discriminating PDAC from benign control patients do not support the conclusion from our pilot study [9]. Reasons for this may include: 1) The smaller control cohort in the pilot study included both benign HPB pathology patients and non-HPB patients, resulting in a control group of patients with varied responses to the phenotype of inflammation; 2) The pilot report used a variety of different assays to quantify the analytes and these were performed in different laboratories, whereas the current study used only multiplex immunoassays in the same laboratory. The advantages of using multiplex assays include the simultaneous evaluation of several analytes from one sample, using minimal sample volume, in a cost- and time-effective manner; 3) statistically the cytokines with values below the limit of detection were dealt with differently in this expanded study and substituted by ½*LOD. Also, where we reported sensitivity and specificity for specific cut-off values of a combined biomarker in our pilot report, we rather report the area under curve and odds ratios of multivariate logistic regressions, enabling a more patient-centred probability of having PDAC in our population when using these methods. Specific cut-off values are assay and often population specific.

This study has a number of limitations. We are limited by our control cohort of patients with benign, non-pancreatic disease, rather than healthy controls. This control group of patients was selected based on their availability of their CT-scans confirming a normal pancreas. Confirming the same in healthy study participants by unnecessary radiation exposure would not be ethically justified. Nevertheless, an advantage of this control cohort is that it allows us to discriminate between diseases with similar phenotypes of inflammation and often similar presenting symptoms, and the significant differences in univariate and multivariate biomarkers between these two groups reduces the limitation of using a non-pancreatic disease control cohort. Another limitation is that some of the biomarkers are low or undetectable in healthy individuals and, therefore, more pronounced differences between our two groups, particularly for angiogenic markers, may be lost when comparing PDAC to non-healthy controls. Moreover, our study is limited by the relatively small number of events in the subgroups to the number of predictors in the model which may produce overfitting of the data. Hence, we need to further validate this study in an independent discovery and validation cohort prior to employing the models in clinical decision making. Finally, the relationship between biomarker panels and prognosis is worthy of investigation, albeit not within the scope of this manuscript.

## Conclusion

In summary, the cytokines CXCL10, IL-8 and IL-15 have emerged as strong biomarkers in discriminating PDAC from other benign diseases in Black South Africans. Specifically, a panel of CXCL10 combined with CA19-9 afforded enhanced diagnostic accuracy in discriminating PDAC patients from patients with benign, non-HPB disease. Furthermore, biomarker panels including IL-8 and/or IL-15 showed significant diagnostic power in discriminating PDAC patients from benign disease patients with biliary pathologies, and continued show diagnostic accuracy in the presence of jaundice or choledocholithiasis. These findings support the value of using combined biomarker panels in establishing or improving diagnostic accuracy in PDAC and further studies are warranted to establish their role in other PDAC populations and better define optimal cut-off levels for these cytokines.

## Supporting information

S1 Table Minimum detectable concentrations of inflammatory cytokines and angiogenic factors in our laboratory. (DOCX)
