# Supplementary material for: Inflammatory cytokines and combined biomarker panels in pancreatic ductal adenocarcinoma: Enhancing diagnostic accuracy
Source: PLoS One. 2019 Aug 15;14(8):e0221169. doi: 10.1371/journal.pone.0221169 (PMC6695103; doi:10.1371/journal.pone.0221169)
Supplement: S1 Table — (DOCX) [file pone.0221169.s001.docx]

**S1 Table. Minimum detectable concentrations of inflammatory cytokines and angiogenic factors in our laboratory.**

| **Cytokines** | **Minimum detectable concentration (pg/mL)** | **Cytokines** | **Minimum detectable concentration (pg/mL)** |
| --- | --- | --- | --- |
| Eotaxin | 1.67 | IL-22 | 0.13 |
| G-CSF | 1.09 | IL-23* | 6.02 |
| GM-CSF | 0.17 | IP-10 | 4.24 |
| IFN-γ | 5.15 | MCP-1 | 2.22 |
| IL-1β | 0.16 | MIP-1α (CCL3) | 0.21 |
| IL-1ra | 1.57 | MIP-1β (CCL4) | 0.61 |
| IL-2* | 0.25 | RANTES (CCL5) | 2.35 |
| IL-4 | 0.13 | TNF-α | 1.81 |
| IL-5 | 0.98 | aFGF | 3.72 |
| IL-6 | 0.49 | bFGF | 7.7 |
| IL-7 | 0.08 | ICAM-1 (ng/mL) | 2627.4 |
| IL-8 | 0.68 | VCAM-1 (ng/mL) | 2704.0 |
| IL-9 | 0.57 | P-selectin (ng/mL) | 72.3 |
| IL-10 | 1.4 | PDGF-AA | 1.38 |
| IL-12 | 1.03 | PDGF-BB | 2.67 |
| IL-13 | 0.38 | PlGF | 0.28 |
| IL-15 | 1.46 | VEGF | 4.8 |
| IL-17A | 3.2 | sVEGF-R2/KDR | 50.9 |
| IL-21* | 0.19 | sVEGF-R1/Flt-1 | 24.35 |

*****Majority of these values were below the limit of detection (LOD) and substituted with ½*LOD.
